# Xanthones from the Leaves of *Garcinia cowa* Induce Cell Cycle Arrest, Apoptosis, and Autophagy in Cancer Cells

**DOI:** 10.3390/molecules200611387

**Published:** 2015-06-19

**Authors:** Zhengxiang Xia, Hong Zhang, Danqing Xu, Yuanzhi Lao, Wenwei Fu, Hongsheng Tan, Peng Cao, Ling Yang, Hongxi Xu

**Affiliations:** 1 School of Pharmacy, Shanghai University of Traditional Chinese Medicine, Shanghai 201203, China; E-Mails: xzx5380537@163.com (Z.X.); zhnjau19851010@163.com (H.Z.); xdq726@126.com (D.X.); laurence_ylao@163.com (Y.L.); fu_wenwei@163.com (W.F.); ths97029@163.com (H.T.); 2 Engineering Research Centre of Shanghai Colleges for TCM New Drug Discovery, Shanghai 201203, China; 3 Jiangsu Province Academy of Traditional Chinese Medicine, No. 100 Shizi Street, Hongshan Road, Nanjing 210028, China; E-Mail: njpcao@126.com; 4 Laboratory of Pharmaceutical Resource Discovery, Dalian Institute of Chemical Physics, Chinese Academy of Sciences, 457 Zhong-shan Road, Dalian 116023, China; E-Mail: yling@dicp.ac.cn

**Keywords:** *Garcinia cowa*, xanthone, cytotoxicity, apoptosis, cell cycle arrest, autophagy

## Abstract

Two new xanthones, cowaxanthones G (**1**) and H (**2**), and 23 known analogues were isolated from an acetone extract of the leaves of *Garcinia cowa*. The isolated compounds were evaluated for cytotoxicity against three cancer cell lines and immortalized HL7702 normal liver cells, whereby compounds **1**, **5**, **8**, and **15**–**17** exhibited significant cytotoxicity. Cell cycle analysis using flow cytometry showed that **5** induced cell cycle arrest at the S phase in a dose-dependent manner, **1** and **16** at the G2/M phase, and **17** at the G1 phase, while **16** and **17** induced apoptosis. Moreover, autophagy analysis by GFP-LC3 puncta formation and western blotting suggested that **17** induced autophagy. Taken together, our results suggest that these xanthones possess anticancer activities targeting cell cycle, apoptosis, and autophagy signaling pathways.

## 1. Introduction

Compounds from natural plants are important sources of drugs against a wide variety of diseases, including cancer. Cell cycle arrest, apoptosis, and autophagy are important signaling pathways during tumorigenesis and chemotherapy [1]. Apoptosis, also called type I programmed cell death (PCD), is mainly controlled by the integrity of the outer membranes of mitochondria and cleavage of a cascade of proteolysis including caspase-3 [2]. Autophagy (type II programmed cell death) is an evolutionarily conserved membrane process that involves initiation, elongation, closure, maturation, and degradation, which are controlled by highly conserved autophagy-related proteins (ATGs) [3,4]. Microtubule-associated protein 1 light chain 3 (LC3) and p62 (SQSTM1/p62) serve as two protein markers for autophagosome formation and autophagic flux [5]. In recent years, high content screening (HCS), which mainly applies imaging based techniques, plays an important role on anticancer drug screen and development. For instance, fluorescent based biosensor such as GFP-LC3 can be used to screen bioactive compounds targeting autophagy [6].

*Garcinia cowa* (Guttiferae) is a tree with edible fruits and leaves found in China in the southern and western parts of Yunnan Province. It contains numerous chemicals with various bioactivities, including anticancer and antibacterial activities. Plants of the genus *Garcinia* have been used as traditional medicines in many countries around the world. Xanthones and polycyclic polyprenylated acylphloroglucinols are characteristic components of the genus *Garcinia* and are well known for their anti-cancer activity. Natural xanthones (dibenzo-γ-pyrones) constitute an important class of oxygenated heterocycles, occurring as secondary metabolites in plants and microorganisms [7]. This type of metabolites exhibits a wide variety of biological activities such as cytotoxicity [8]; monoamine oxidase inhibition [9]; and antioxidant [10], antimicrobial [11], antiviral, antifungal [12], hepatoprotective [13], antithrombotic [14], and antiinflammatory activities [15]. In recent years, the fascinating chemical structures and biological activities of xanthones have attracted widespread attention from phytochemists [16], synthetic organic chemists [17,18,19], and pharmacologists [20,21,22].

**Figure 1:**
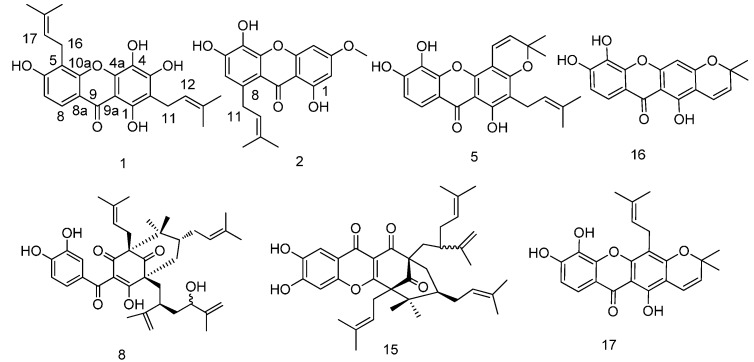
New and active compounds from *G. cowa*.

In this study, we identified and analyzed the bioactivities of the xanthones on cell cycle, apoptosis, and autophagy from *Garcinia cowa*. Bioassay-directed fractionation of *G. cowa* yielded two new xanthones called cowaxanthones G and H (**1** and **2**, Figure 1), and 23 known derivatives. Herein, we report the isolation, structure elucidation, and bioactivities of these compounds.

## 2. Results and Discussion

The leaves of *G. cowa* were pulverized and extracted three times with acetone at room temperature. The acetone extract was suspended in hot water and partitioned with CH_2_Cl_2_. The CH_2_Cl_2_-soluble portion was subjected to repeated chromatography over silica gel, reversed-phase C_18_ silica gel, and preparative HPLC to afford 25 pure compounds (>95% as evidenced by the ^1^H- and ^13^C-NMR spectra as well as HPLC analyses).

Compound **1** was shown to have the molecular formula C_23_H_24_O_6_ by HRESIMS measurement (*m*/*z*, 395.1486 [M − H]^−^). The ^13^C-NMR and DEPT disclosed the presence of a carbonyl, 12 *sp*^2^ quaternary carbons (six of which were oxygenated), four sp^2^ methine, two sp^3^ methylene, and four methyl groups. The ^1^H-NMR spectrum exhibited a signal for a chelated hydroxylic proton at δ_H_ 13.46 (s) and two *ortho*-aromatic protons at δ_H_ 7.49 (1H, d, *J* = 8.6 Hz) and 6.90 (1H, d, *J* = 8.6 Hz) (Table 1).

**Table 1:** ^1^H- and ^13^C-NMR spectroscopic data of **1** and **2**.

No	1 ^a^		2 ^a^	
	δ_C_	δ_H_ (*J* in Hz)	δ_C_	δ_H_ (*J* in Hz)
1	161.2		163.1	
2	110.6		96.8	6.30, d (1.9)
3	158.1		165.7	
4	132.9		96.8	6.51, d (1.9)
4a	152.8		156.6	
5	107.0		131.1	
6	152.7		151.9	
7	113.3	6.90, d (8.6)	114.4	6.70, s
8	116.3	7.49, d (8.6)	134.7	
8a	101.4		110.4	
9	180.4		182.1	
9a	101.7		-	
10a	146.8		156.6	
11	21.7	3.56, d (6.8)	33.3	3.85, m
12	122.8	5.23, t (6.8)	123.4	5.30, t (6.6)
13	131.0		131.7	
14	18.1	1.81, s	25.8	1.68, s
15	25.7	1.62, s	18.0	1.68, s
16	21.5	3.31, d (6.8)		
17	123.0	5.16, t (6.8)		
18	130.0			
19	18.0	1.74, s		
20	25.8	1.63, s		
1-OH		13.46, s		13.52, s
3-OMe			56.1	3.85, s

^a^ Recorded at 400 MHz (^1^H) and 100 MHz (^13^C) in DMSO-*d*_6_.

**Figure 2:**
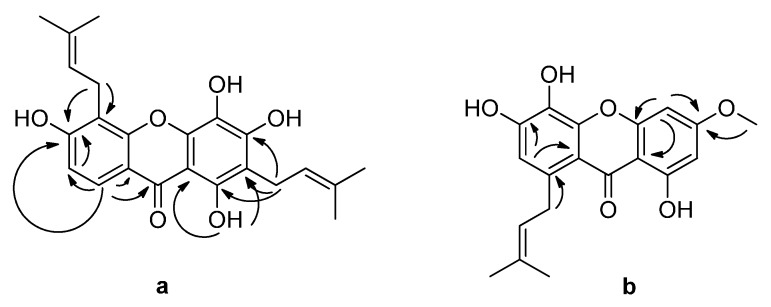
Key HMBC (H→C) correlations of (**a**) **1** and (**b**) **2**.

In addition, two olefinic protons at δ_H_ 5.23 (1H, t, *J* = 6.8 Hz) and 5.16 (1H, t, *J* = 6.8 Hz), four olefinic methyl signals at δ_H_ 1.81 (3H, s), 1.74 (3H, s), 1.63 (3H, s), and 1.62 (3H, s), and four allylic protons at δ_H_ 3.56 (2H, d, *J* = 6.8 Hz) and 3.31 (2H, d, *J* = 6.8 Hz) were observed in the ^1^H-NMR spectrum, indicating the existence of two prenyl groups in **1**. The two prenyl moieties were located at C-2 (δ_C_ 110.6) and C-5 (δ_C_ 107.0) based on HMBC correlations (Figure 2). Thus, **1** was determined to be 1,3,4,6-tetrahydroxy-2,5-di(3-methylbut-2-enyl)-xanthone, and was named cowaxanthone G.

Compound **2** was isolated as a yellow gum. The molecular formula C_19_H_18_O_6_ was deduced by HRESIMS at *m*/*z* 341.1015 [M − H]^−^. The ^1^H-NMR spectrum (Table 1) exhibited signal of a methoxy group at δ_H_ 3.85 (3H, s) and three aromatic protons: H-2 at δ_H_ 6.30 (1H, d, *J* = 1.9 Hz), H-4 at δ_H_ 6.51 (1H, d, *J* = 1.9 Hz), and H-7 at δ_H_ 6.70 (1H, s). Also observed was an isoprene moiety with a pair of *gem*-dimethyl signals at δ_H_ 1.68 (6H, s), a methine signal at δ_H_ 5.30 (1H, t, *J* = 6.6 Hz), and a methylene signal at δ_H_ 3.85 (2H, m). The ^13^C-NMR spectrum displayed 19 peaks (Table 1), including one carbonyl, two aromatic rings with oxygenated carbons, a methoxy group and one isoprene moiety. These signals implied a trihydroxylated xanthone with methoxy and isoprene groups. The isoprene moiety was attached at C-8 (δ_C_ 134.7) and the methoxy group at C-3 (δ_C_ 165.7) based on HMBC correlations (Figure 2). Compound **2**, which was named cowaxanthone H, was thus identified as 1,5,6-trihydroxy-3-methoxy-8-(3-methylbut-2-enyl)xanthone.

The known compounds isojacareubin (**3**) [23], 1,3,5-trihydroxy-6′,6′-dimethyl-2*H*-pyrano-(2′,3′:6,7)xanthone (**4**) [24], 1,5,6-trihydroxy-2-prenyl-6′,6′-dimethyl-2*H*-pyrano(2′,3′:3,4)xanthone (**5**) [25], dulxanthone A (**6**) [26], cambogin (**7**), garcimultiflorones E and F (compounds **8** and **9**) [27], oblongifolin C (**10**) [28], guttiferone F (**11**) [29], garciniagifolone A (**12**) [30], garcicowins C and D (**13** and **14**) [31], symphonone H (**15**), jacareubin (**16**) [32], xanthone V_1_ (**17**) [33], isoprenylxanthone (**18**) [34], garcinexanthone C (**19**) [35], xanthone V_1a_ (**20**) [33], 1,3,5-trihydroxyxanthone (**21**) [36], ugaxanthone (**22**) [37], 1,5,6-trihydroxy-3-methoxyxanthone (**23**) [38], 1,3,7-trihydroxylxanthone (**24**) [39], and 1,4,5-trihydroxyxanthone (**25**) [19] were identified by comparison of their spectroscopic data with reported values.

All isolated compounds **1**–**25** were evaluated for cytotoxicity against three human cancer cell lines (HeLa, PANC-1, and A549) and selectivity determined by using immortalized HL7702 normal human liver cells. The anticancer drug etoposide was used as positive control. Compounds **1**, **5**, **8**, **15**–**17** showed significant inhibition on cell viability (IC_50_ < 10 μM, Table 2).

**Table 2:** Cytotoxicity of compounds against three cancer cell lines *^a^*.

Compounds	HeLa	A549	PANC-1	HL-7702
**1**	8.09 ± 0.78	12.57 ± 4.30	14.80 ± 8.68	11.00 ± 4.36
**5**	7.06 ± 0.71	8.19 ± 0.99	9.32 ± 4.58	10.45 ± 4.122
**8**	17.61 ± 1.45	7.57 ± 0.57	17.73 ± 1.56	7.34 ± 0.65
**15**	9.83 ± 0.61	6.27 ± 0.71	11.24 ± 4.89	3.96 ± 2.38
**16**	1.09 ± 0.67	6.90 ± 2.23	10.12 ± 7.91	5.50 ± 1.79
**17**	4.71 ± 0.52	11.76 ± 6.29	6.56 ± 2.55	9.50 ± 3.74
**Etoposide *^b^***	2.91 ± 0.16	1.31 ± 0.09	22.76 ± 1.93	1.42 ± 0.13

*^a^* Results are expressed as IC_50_ values in μM. *^b^* Positive control.

**Figure 3:**
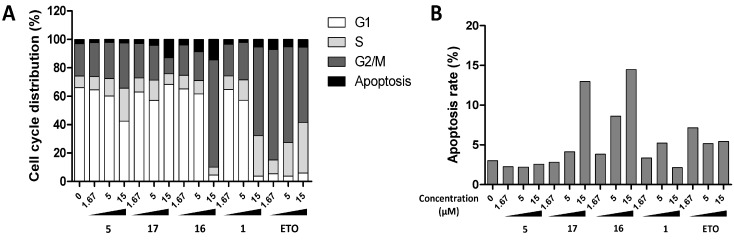
Effects of **1**, **5**, **16**, and **17** on cell cycle and apoptosis. HeLa cells were treated with indicated chemicals. After 24 h of treatment, the cells were harvested, fixed in 70% EtOH, and stained with PI. The cell cycle and apoptosis rate were detected by FACS. Etoposide is used as positive control; (**A**) Cell cycle attribution of HeLa cells under different concentration treatment; (**B**) Apoptotic rates were acquired from Sub-G1 fraction from A.

**Figure 4:**
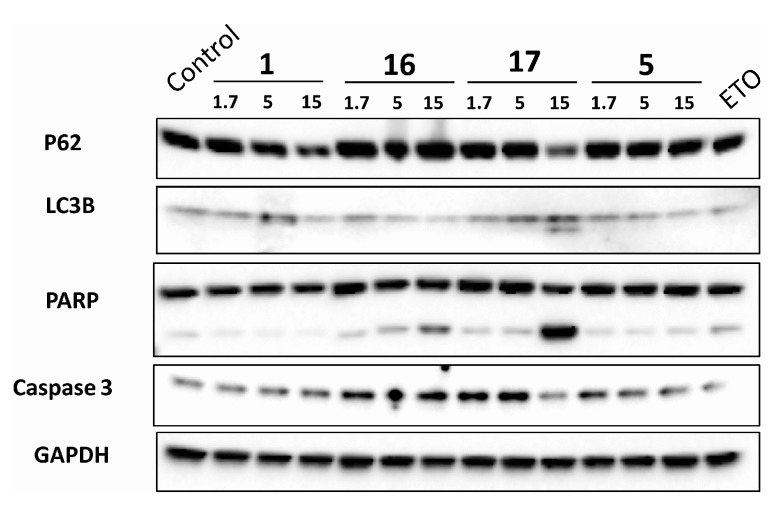
Effects of **1**, **5**, **16**, and **17** on apoptosis and autophagy related proteins. Western blots of p62, LC3B, PARP, Caspase 3, and GAPDH of HeLa cells after 24 h treatment with **1**, **5**, **16**, and **17**. Etoposide was used as positive control.

**Figure 5:**
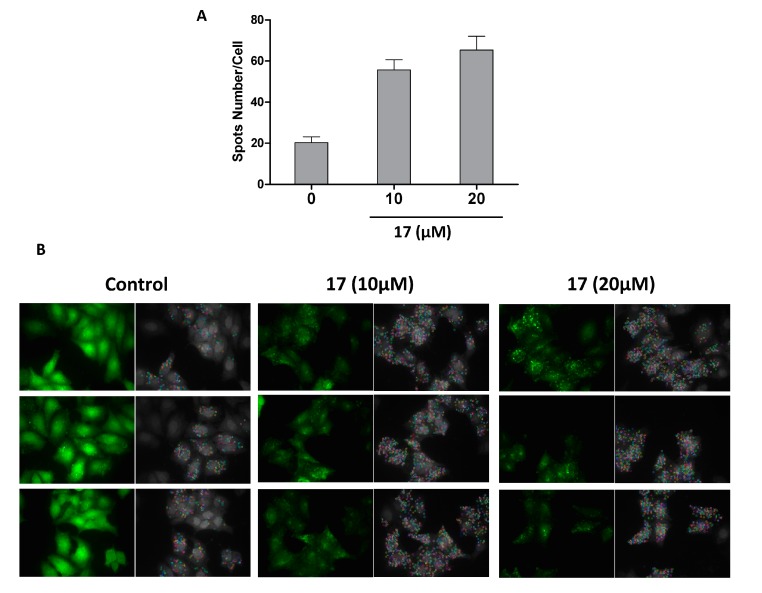
Xanthone V_1_ (**17**) induces GFP-LC3 puncta formation in HeLa cells. HeLa cells stably expressing GFP-LC3 were seeded in a 96-well dish and treated with 10, 20 µM **17** for 24 h. (**A**) The number of GFP-LC3 puncta in each samples were calculated by Columbus software. The number was averaged of cells from three wells; (**B**) The images of GFP-LC3 puncta after 10, 20 µM treatment Xanthone V_1_ were acquired by an Opera (GFP ex and em) with a 40×-H_2_O objective. Left Panel: the original GFP-LC3 puncta; Right panel: the spots were auto-analyzed by Columbus software.

Based on their potency and selectivity for the cancer cells, compounds **1**, **5**, **16**, and **17** were selected as potential chemotherapeutic compounds, and further investigation of their mechanism of action was undertaken. We first evaluated their effects on apoptosis and cell cycle arrest by flow cytometry. We found (Figure 3) that **5** induced cell cycle arrest at the S phase in a dose-dependent fashion, **1** and **16** at the G2/M phase, and **17** at the G1 phase, while **16** and **17** induced apoptosis (Figure 3). Next, we performed western blot analysis of key proteins mediating apoptosis and autophagy, including caspase-3, PARP, LC3B, and p62 (Figure 4). **5**, **16**, and **17** activated PARP cleavage, suggesting that they activate apoptosis. **17** increased the conversion of LC3-I to LC3-II and p62 reduction, suggesting that **17** may promote autophagy. To confirm this, we examined GFP-LC3 puncta formation in HeLa cells after treatment with **17**. As shown in Figure 5, an increase in GFP-LC3 puncta was observed by confocal images.

## 3. Experimental Section

### 3.1. General

Optical rotations were measured with a SEPA-300 polarimeter (Horiba, Kyoto, Japan). Ultraviolet absorption spectra were recorded on a UV-2401 PC spectrophotometer (Shimadzu Corporation, Kyoto, Japan). IR spectra were obtained using an FtS-135 spectrometer (Bio-Rad, Hercules, CA, USA). NMR spectra were measured on an AV-400/500/600 spectrometer (Bruker, Baden-Württemberg, Germany) with TMS as internal standard. Mass spectrometry was performed on a Q-TOF Premier instrument (Waters MS Technologies, Manchester, UK) equipped with an ESI source. Column chromatography was performed with silica gel (200–300 mesh) (Qingdao Haiyang Chemical Co., Ltd., Qingdao, China) and reversed-phase C_18_ silica gel (50 μm, YMC, Kyoto, Japan). Precoated sheets of silica gel 60 GF_254_ were used for TLC. A Waters 2535 Series machine with Xbridge C_18_ column17 (4.6 × 250 mm, 5 μm) was used for HPLC. A preparative Xbridge Prep C_18_ OBD column (19 × 250 mm, 5 μm) was used for sample preparation (Waters Corporation, Milford, MA, USA). Etoposide (purity > 98%) was purchased from Sigma-Aldrich Trading Co. Ltd. (Shanghai, China).

### 3.2. Plant Material

Leaves of *Garcinia cowa* were collected in Xishuangbanna, Yunnan Province, People’s Republic of China, in August 2012. The plant material was identified by Prof. Hong Wang, Xishuangbanna Tropical Botanical Garden, Chinese Academy of Sciences. A voucher specimen (G. C. 0001) was deposited in the School of Pharmacy, Shanghai University of Traditional Chinese Medicine.

### 3.3. Extraction and Isolation

Leaves of *G. cowa* (3.5 kg) were pulverized and extracted with acetone (3 × 20 L, each two days). at room temperature. The acetone extract (160 g) was suspended in hot water (2.5 L) and partitioned against CH_2_Cl_2_ (5 × 3 L). The CH_2_Cl_2_-soluble fraction (36 g) was then subjected to silica gel column chromatography (8 × 80 cm; 200–300 mesh; 1000 g) and eluted with a CH_2_Cl_2_–MeOH gradient (1:0 to 0:1, *v*/*v*; 1000 mL each) to afford five fractions (A–E), as monitored by TLC. Fractions A, B, and C showed cytotoxicity against HeLa, PANC-1, and A549 tumor cell lines. Fraction A (5 g) was chromatographed over a column of silica gel (4 × 45 cm; 200–300 mesh; 250 g) and eluted with petroleum ether–ethyl acetate (3:1, *v*/*v*; 100 mL each) to give four subfractions (A1–A3). Of these, subfraction A2 (32 mg) was subjected to chromatography on reversed-phase C_18_ silica gel (1.5 × 15 cm; 20 g), using methanol–water (9:1) as the mobile phase, and then further purified by preparative HPLC using acetonitrile–water (88:12, 0.1% trifluoroacetic acid, 16 mL/min) as the mobile phase to give **10** (15 mg) and **15** (2 mg). Fraction B (12 g) was chromatographed over reversed-phase C_18_ silica gel (6 × 50 cm; 600 g) and eluted with a methanol–water gradient (80:20 to 95:5, *v*/*v*; 500 mL each) to give four subfractions (B1–B4). Subfraction B1 (8 mg) was separated by preparative HPLC using acetonitrile–water (60:40, 0.1% trifluoroacetic acid, 16 mL/min) as the mobile phase to yield **8** (5 mg) and **9** (2 mg). Subfraction B2 (15 mg) was separated by preparative HPLC using acetonitrile–water (62:38, 0.1% trifluoroacetic acid, 16 mL/min) as the mobile phase to yield **7** (3 mg), **13** (3 mg), and **14** (2 mg). Subfraction B3 (206 mg) was separated by preparative HPLC using acetonitrile–water (65:35, 0.1% trifluoroacetic acid, 16 mL/min) as the mobile phase to give **11** (5 mg) and **12** (6 mg). Fraction C (14 g) was chromatographed over reversed-phase C_18_ silica gel and eluted with a methanol–water gradient (45:55 to 90:10) to give five subfractions (C1–C5). Subfraction C1 (63 mg) was separated by preparative HPLC using acetonitrile–water (20:80, 0.1% trifluoroacetic acid, 16 mL/min) as the mobile phase to yield **21** (5 mg), **24** (4 mg), and **25** (10 mg). Subfraction C2 (63 mg) was separated by preparative HPLC using acetonitrile–water (23:77, 0.1% trifluoroacetic acid, 16 mL/min) as the mobile phase to yield **16** (5 mg), **1** (5 mg), and **2** (10 mg). Subfraction C3 (63 mg) was separated by preparative HPLC using acetonitrile–water (25:75, 0.1% trifluoroacetic acid, 16 mL/min) as the mobile phase to yield **17** (5 mg), **23** (5 mg), and **6** (10 mg). Subfraction C4 (63 mg) was separated by preparative HPLC using acetonitrile–water (30:70, 0.1% trifluoroacetic acid, 16 mL/min) as the mobile phase to yield **18** (5 mg), **19** (5 mg), and **22** (5 mg). Subfraction C5 (63 mg) was separated by preparative HPLC using acetonitrile–water (35:65, 0.1% trifluoroacetic acid, 16 mL/min) as the mobile phase to yield **1** (5 mg), **2** (5 mg), **5** (5 mg), and **20** (5 mg).

### 3.4. Characterization

*Cowaxanthone G* (**1**). Yellow gum; UV (MeOH) λmax (log ε) 327 (3.20), 253 (4.61) nm; IR (KBr) νmax 3419, 2919, 1649, 1608, 1585, 1284, 1207, 1159 cm^−1^; ^1^H- and ^13^C-NMR, see Table 1; HRESIMS *m*/*z* 395.1486 [M − H]^−^ (calcd for C_23_H_23_O_6_, 395.1495).

*Cowaxanthone H* (**2**). Yellow gum; UV (MeOH) λmax (log ε) 327 (3.21), 253 (4.57) nm; IR (KBr) νmax 3427, 2972, 2923, 1639, 1600, 1288 cm^−1^; ^1^H- and ^13^C-NMR, see Table 1; HRESIMS *m*/*z* 341.1015 [M − H]^−^ (calcd for C_19_H_17_O_6_, 341.1025).

### 3.5. Cytotoxicity Assay

The CCK-8 assay was used to determine cell viability. Test samples were dissolved in dimethyl sulfoxide (DMSO) to make stock solutions and further diluted in culture medium for assays. Human cancer cell lines (HeLa, PANC-1, and A549) were cultured in RPMI 1640, DMEM or DMEM/F12 (1:1) medium containing 10% fetal bovine serum. Cells were maintained at 37 °C in a humidified environment under 5% CO_2_. Cell proliferation assays were performed as previously described [40]. Briefly, each cell line was seeded in a 96-well tissue culture plate at a predetermined density in 180 µL of complete medium, attached overnight and treated with test compound for 72 h. Then the medium was discarded and replaced with 10% CCK-8 in complete medium and the plates incubated for another 2 h. OD450 was measured with a SpectraMAX 190 spectrophotometer (MDS, Sunnyvale, CA, USA). Background absorbance was subtracted for all wells. Inhibition rate (IR) was determined. IR (%) = (OD_DMSO_ − OD_compound_)/OD_DMSO_ × 100%.

### 3.6. Flow Cytometry Analysis of Apoptosis and Cell Cycle Arrest

Hypodiploid DNA and cell cycle arrest were evaluated as described previously. Briefly, after treatment of HeLa cancer cells with vehicle (0.1% DMSO) or test compound at the indicated concentrations and times, the cells were harvested by trypsinization and fixed with 70% (*v*/*v*) alcohol at 4 °C for 30 min. After washing with PBS, RNase (10 µg/mL) was added and incubated for 15 min at 37 °C to eliminate RNA interference. The cells were then treated with propidium iodide (PI) for another 30 min. The cells were washed, and the DNA content determined using FACSCalibur [41].

### 3.7. Western Blot Analysis

Cell lysate was prepared in RIPA buffer and quantified by the bicinchoninic acid (BCA) method (Pierce, Rockford, IL, USA). Thirty micrograms of protein per sample was loaded onto a 4%~12% NuPAGE^®^ Novex SDS gel. Protein was transferred using an iBlot^®^ dry blotting device onto nitrocellulose membranes. After blocking nonspecific binding with TBS/Tween20 (0.1%) (TBS/T) containing 5% non-fat milk for 1 h at room temperature, the membrane was incubated in LC3B (Sigma, L7543), SQSTM1/p62 (MBL, PM045), PARP (CST #9542P, Cell Signaling Technology, Shanghai, China), or caspase-3 (CST #9662P, Cell Signaling Technology) (1:1000 in TBS/T containing 3% bovine serum albumin (BSA) in all cases) with gentle shaking at 4 °C overnight. Membranes were washed three times with TBS/T to remove unbound antibody and then incubated with secondary antibody (HRP-conjugated goat anti-mouse IgG or goat anti-rabbit IgG (1:5000) for 1 h at room temperature. Protein bands were visualized with an enhanced chemiluminescence kit (ECL, Pierce).

### 3.8. GFP-LC3 Imaging

HeLa cells stably expressing GFP-LC3 were generated as previously described [6]. Briefly, HeLa cells were transfected with pEGFP-LC3 plasmid using Lipofectamine 2000 (11668-019, Invitrogen, Carlsbad, CA, USA). One day after transfection, the cells were treated with 800 μg/mL G418 for 7 days. Surviving cells (termed HeLa-GFP-LC3 below) were continually cultured with 800 μg/mL G418. HeLa-GFP-LC3 cells were seeded in a 96-well plate overnight (clear bottom, black, Perkin-Elmer, Waltham, MA, USA). The cells were then treated with test compounds in triplicate. After 48 h, the cells were fixed with 4% paraformaldehyde and washed three times with PBS. Image acquisition was performed using an Opera High Content Screening System (Perkin-Elmer) using a 40×-H_2_O objective. The data were analyzed using Columbus 2.3 software (Perkin-Elmer). To quantify GFP-LC3 spots, the following procedures were performed: 1. Define the nuclear region using the Hoechst channel (method A; common threshold 0.45 with area > 100 µM^2^), 2. Define the cellular cytoplasm using the GFP channel (method A; individual threshold 0.15), 3. Calculate spot size in each cell’s exclusive nuclear region (Method C; Radius ≤ 5.0 µM; Contrast > 0.13; Spot to region intensity > 1.0; Distance ≥ 2.3 px; Peak Radius 1.0 px).

## 4. Conclusions

We have isolated two new compounds, cowaxanthones G (**1**) and H (**2**), and 23 known analogues from the leaves of *Garcinia cowa*. These compounds were fully characterized by NMR and HR-MS. Among a multitude of bioactive findings, compounds **1**, **5**, **16** and **17** show strong activities on arresting cell cycle, inducing apoptosis, and activating autophagy. Our findings indicate that *G. cowa* extracts have anticancer potential. In addition, compounds **1**, **5**, **16** and **17** could be used as lead compounds for the development of anticancer drugs. It will be interesting to further explore the detailed mechanism of action of these compounds.

## Supplementary Materials

Supplementary materials can be accessed at: http://www.mdpi.com/1420-3049/20/06/11387/s1.
