# Supplementary material for: Xanthones from the Leaves of Garcinia cowa Induce Cell Cycle Arrest, Apoptosis, and Autophagy in Cancer Cells
Source: Molecules. 2015 Jun 19;20(6):11387–99. doi: 10.3390/molecules200611387 (PMC6272390; doi:10.3390/molecules200611387)
Supplement: Supplementary file 1 [file molecules-20-11387-s001.pdf]

Supplementary Materials

List of supporting information

Figure S1. ESIMS spectrum of **1**.
Figure S2. HRESIMS spectrum of **1**.
Figure S3. IR spectrum of **1**.
Figure S4. ^{1}H-NMR spectrum (400 MHz, DMSO-d_6) of **1**.
Figure S5. ^{13}C-NMR spectrum (100 MHz, DMSO-d_6) of **1**.
Figure S6. HMBC spectrum (400 MHz, DMSO-d_6) of **1**.
Figure S7. HSQC spectrum (400 MHz, DMSO-d_6) of **1**.
Figure S8. ESIMS spectrum of **2**.
Figure S9. HRESIMS spectrum of **2**.
Figure S10. IR spectrum of **2**.
Figure S11. ^{1}H-NMR spectrum (400 MHz, DMSO-d_6) of **2**.
Figure S12. ^{13}C-NMR spectrum (100 MHz, DMSO-d_6) of **2**.
Figure S13. HMBC spectrum (400 MHz, DMSO-d_6) of **2**.
Figure S14. HSQC spectrum (400 MHz, DMSO-d_6) of **2**.
Figure S15. Flow cytometric analysis of HeLa cells. HeLa cells were treated with cisplatin or compound: control (A), 1.67 μM **3** (B), 5 μM **3** (C), 15 μM **3** (D) for 24 h.
Figure S16. Flow cytometric analysis of HeLa cells. HeLa cells were treated with cisplatin or compound: control (A), 1.67 μM **1** (B), 5 μM **1** (C), 15 μM **1** (D) for 24 h.
Figure S17. Flow cytometric analysis of HeLa cells. HeLa cells were treated with cisplatin or compound: control (A), 1.67 μM Xanthone V_1 (B), 5 μM Xanthone V_1 (C), 15 μM Xanthone V_1 for 24 h.
Figure S18. Flow cytometric analysis of HeLa cells. HeLa cells were treated with cisplatin or compound: control (A), 1.67 μM jacareubin (B), 5 μM jacareubin (C), 15 μM jacareubin (D) for 24 h.
Figure S19. Flow cytometry analysis of HeLa cells. HeLa cells were treated with cisplatin or compound: control (A); 1.67 μM etoposide (B); 5 μM etoposide (C); 15 μM etoposide (D) for 24 h.

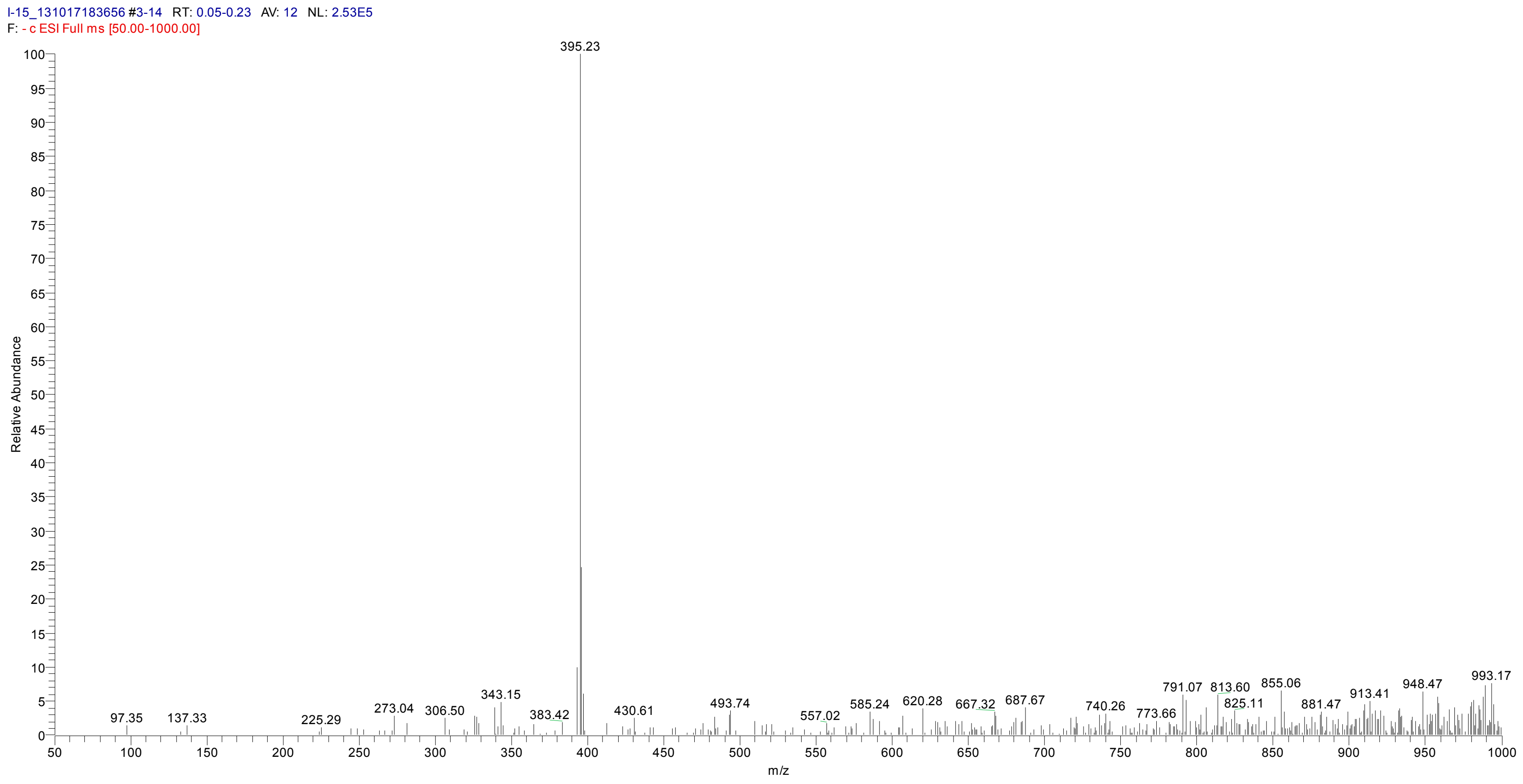

Figure S1. ESIMS spectrum of **1**.

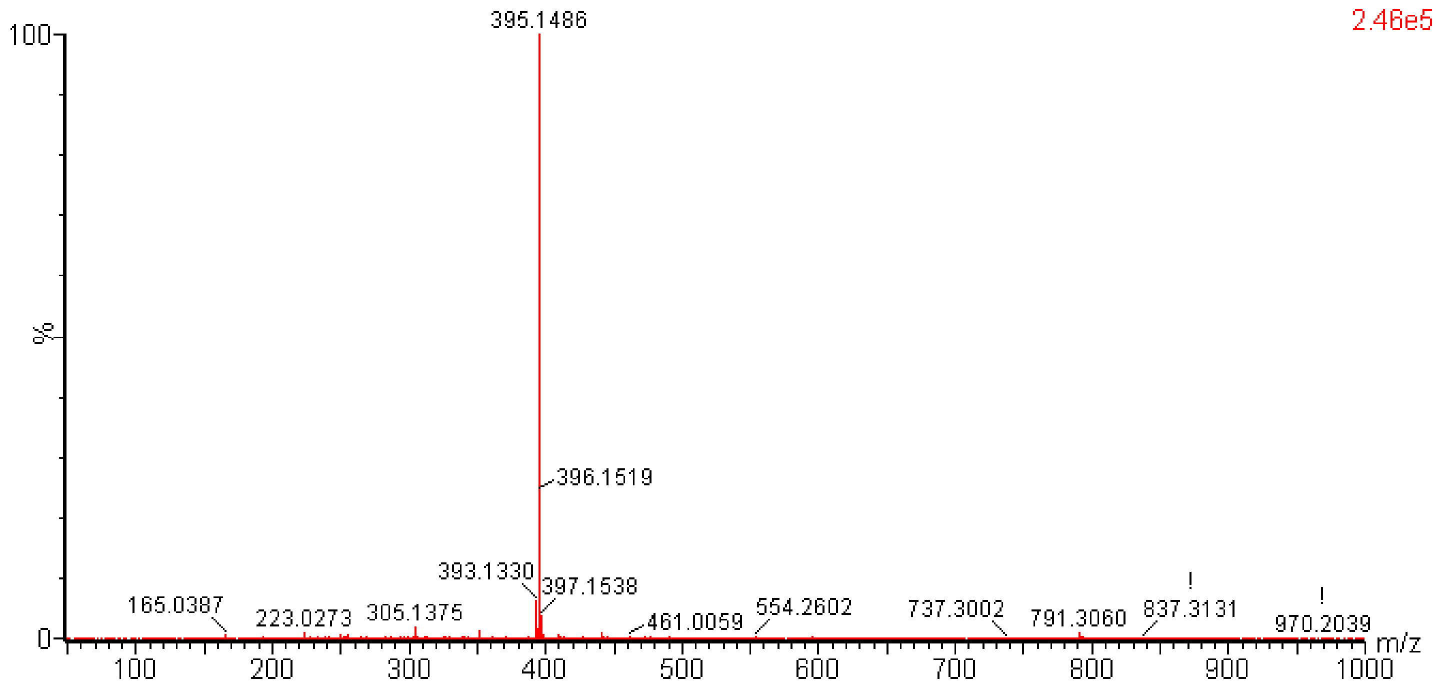

Figure S2. HRESIMS spectrum of **1**.

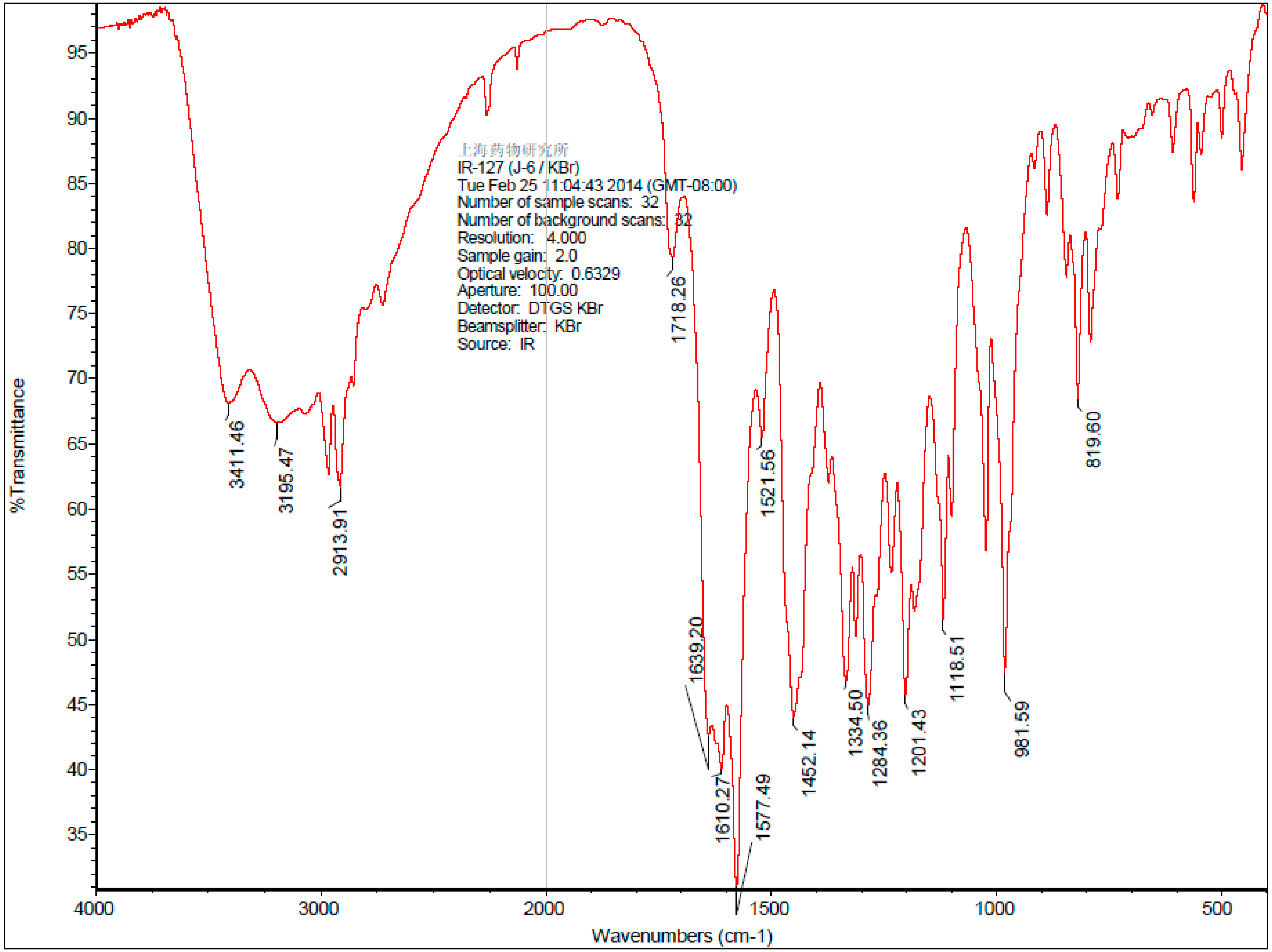

Figure S3. IR spectrum of **1**.

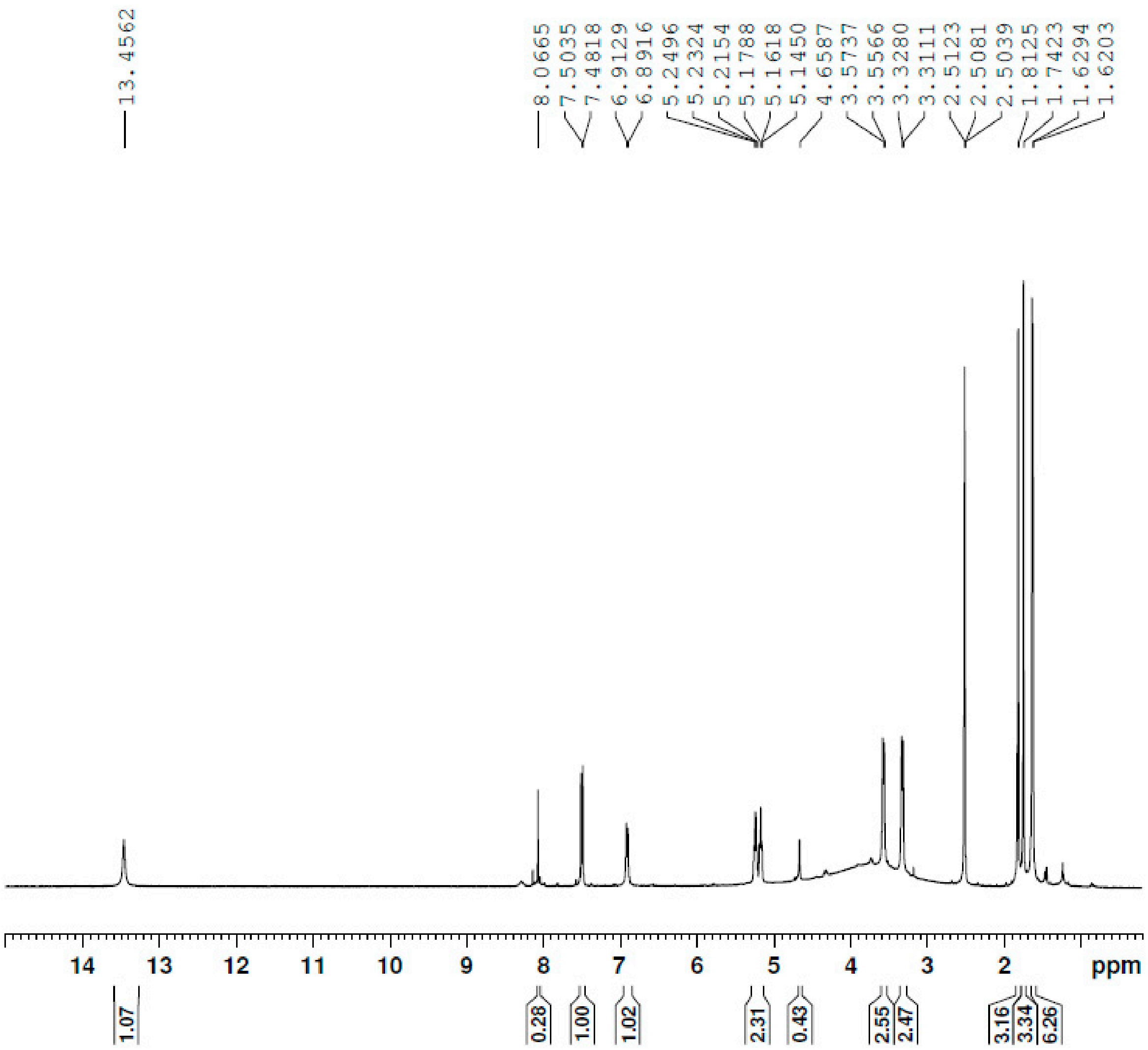

Figure S4. ^{1}H-NMR spectrum (400 MHz, DMSO-d_6) of **1**.

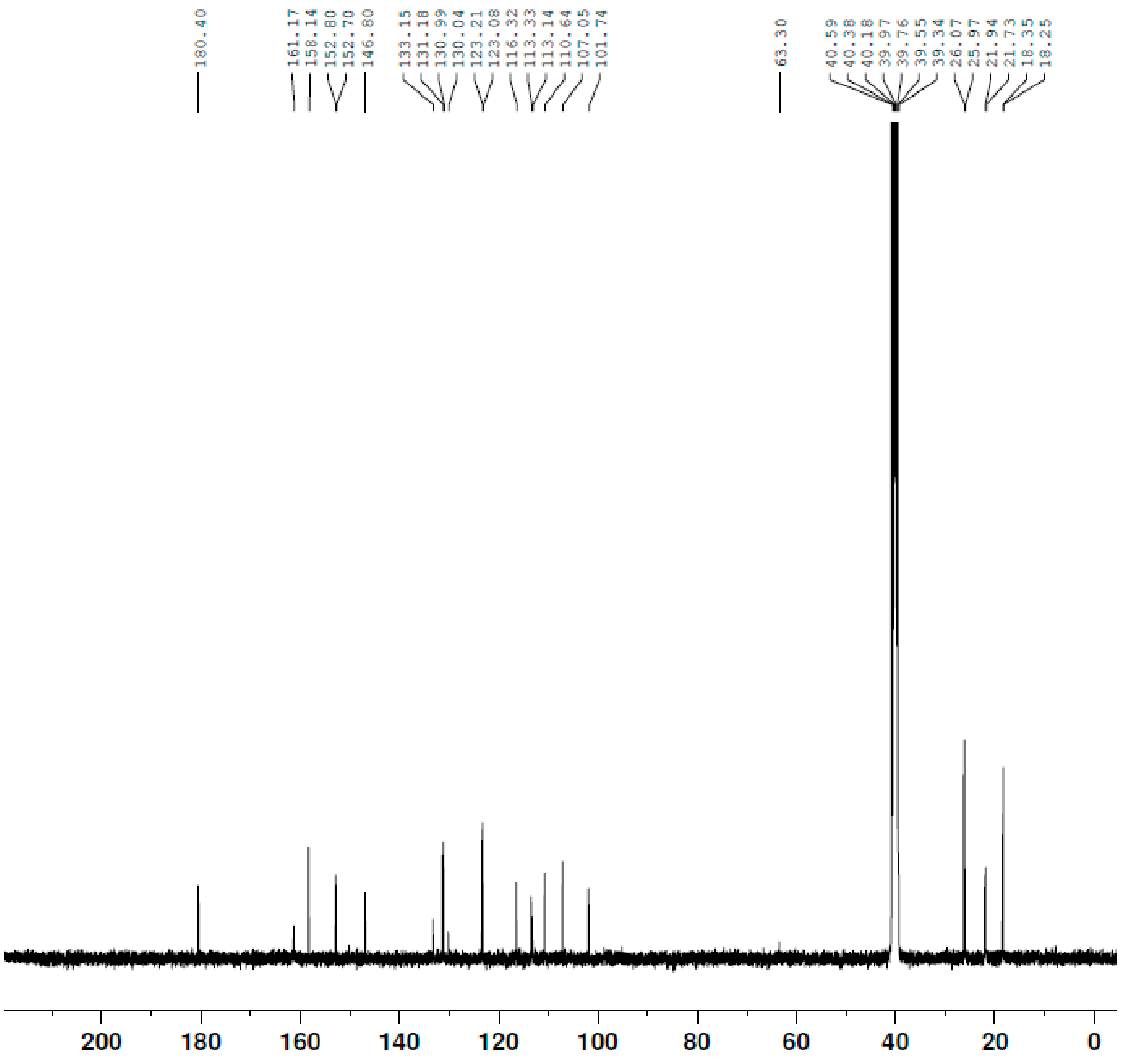

Figure S5. ^{13}C-NMR spectrum (100 MHz, DMSO-d_6) of **1**.

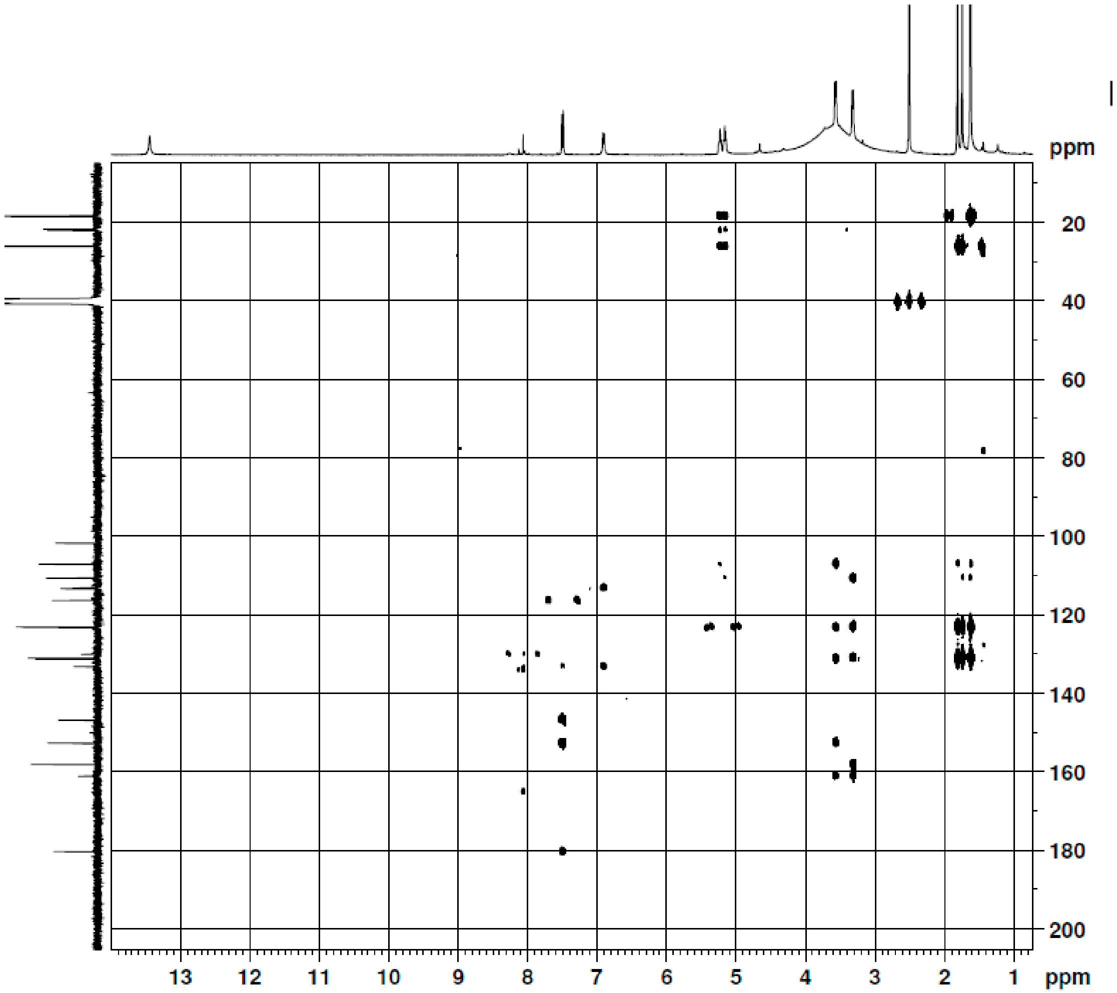

Figure S6. HMBC spectrum (400 MHz, DMSO-d_6) of **1**.

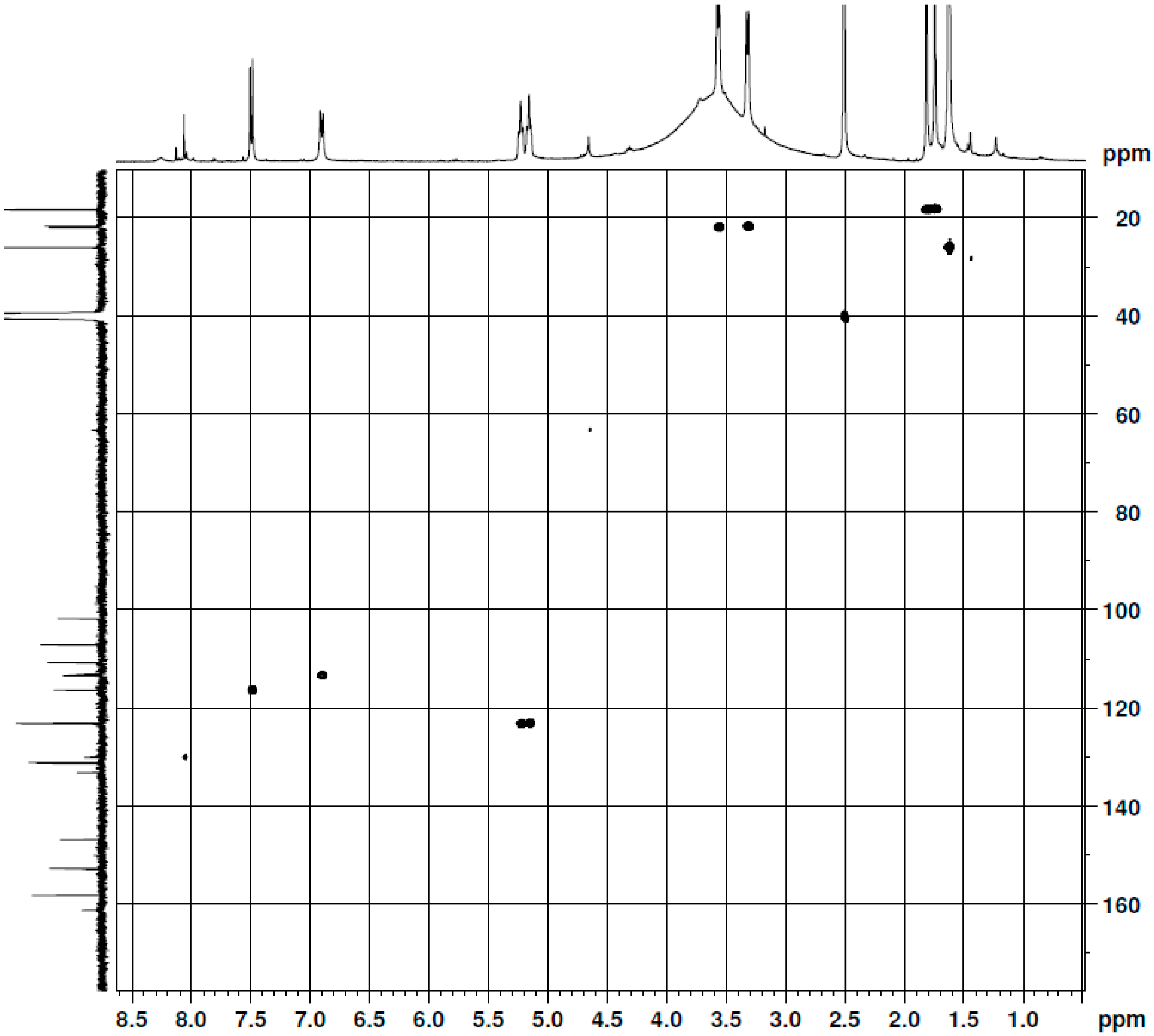

Figure S7. HSQC spectrum (400 MHz, DMSO-d_6) of **1**.

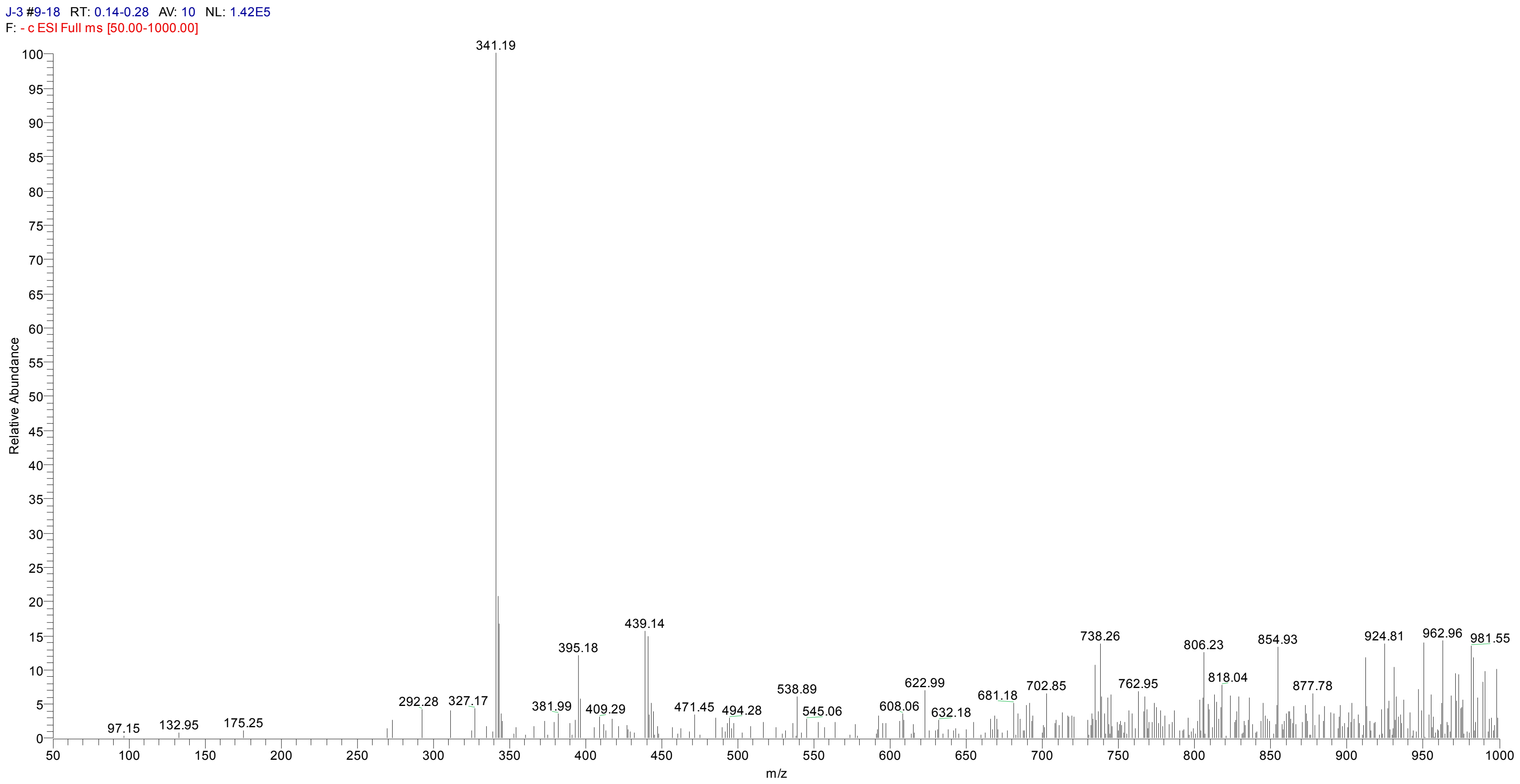

Figure S8. ESIMS spectrum of **2**.

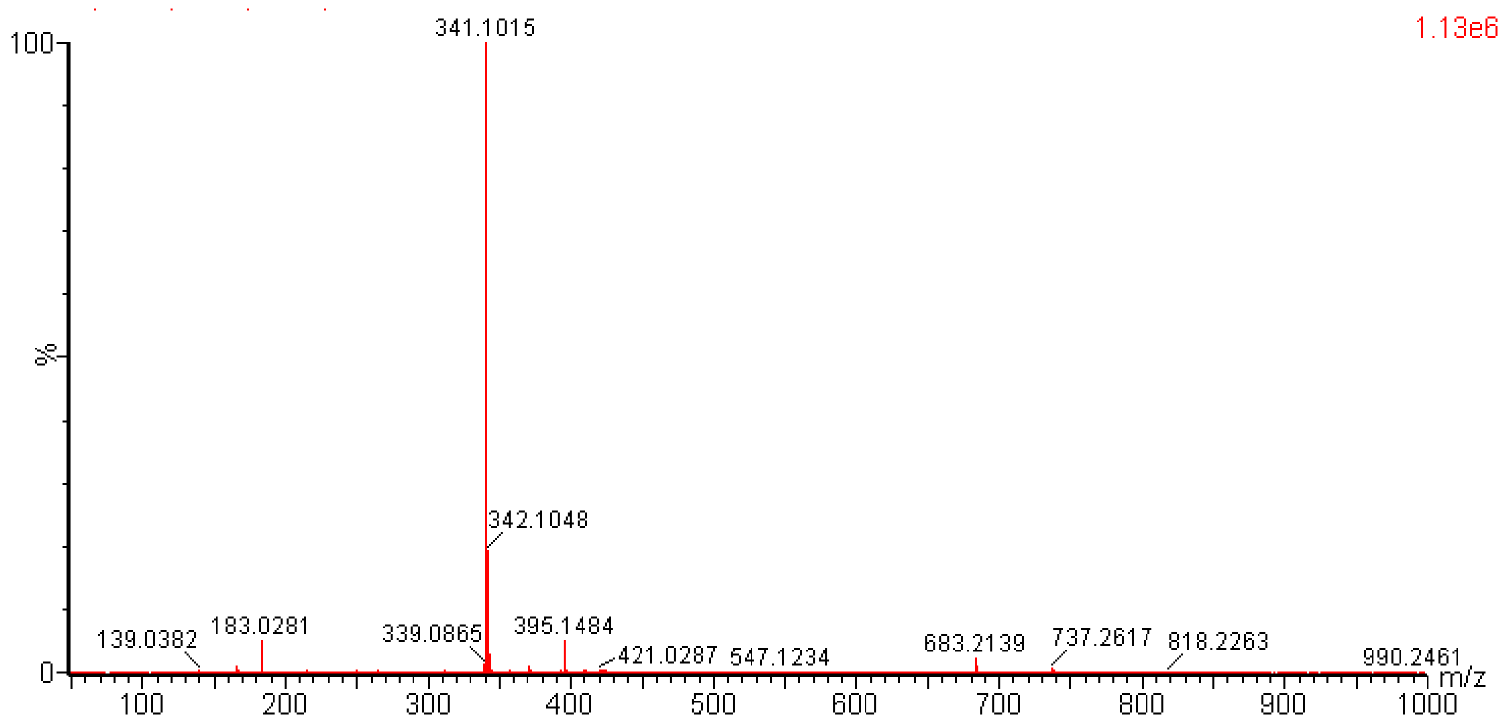

Figure S9. HRESIMS spectrum of **2**.

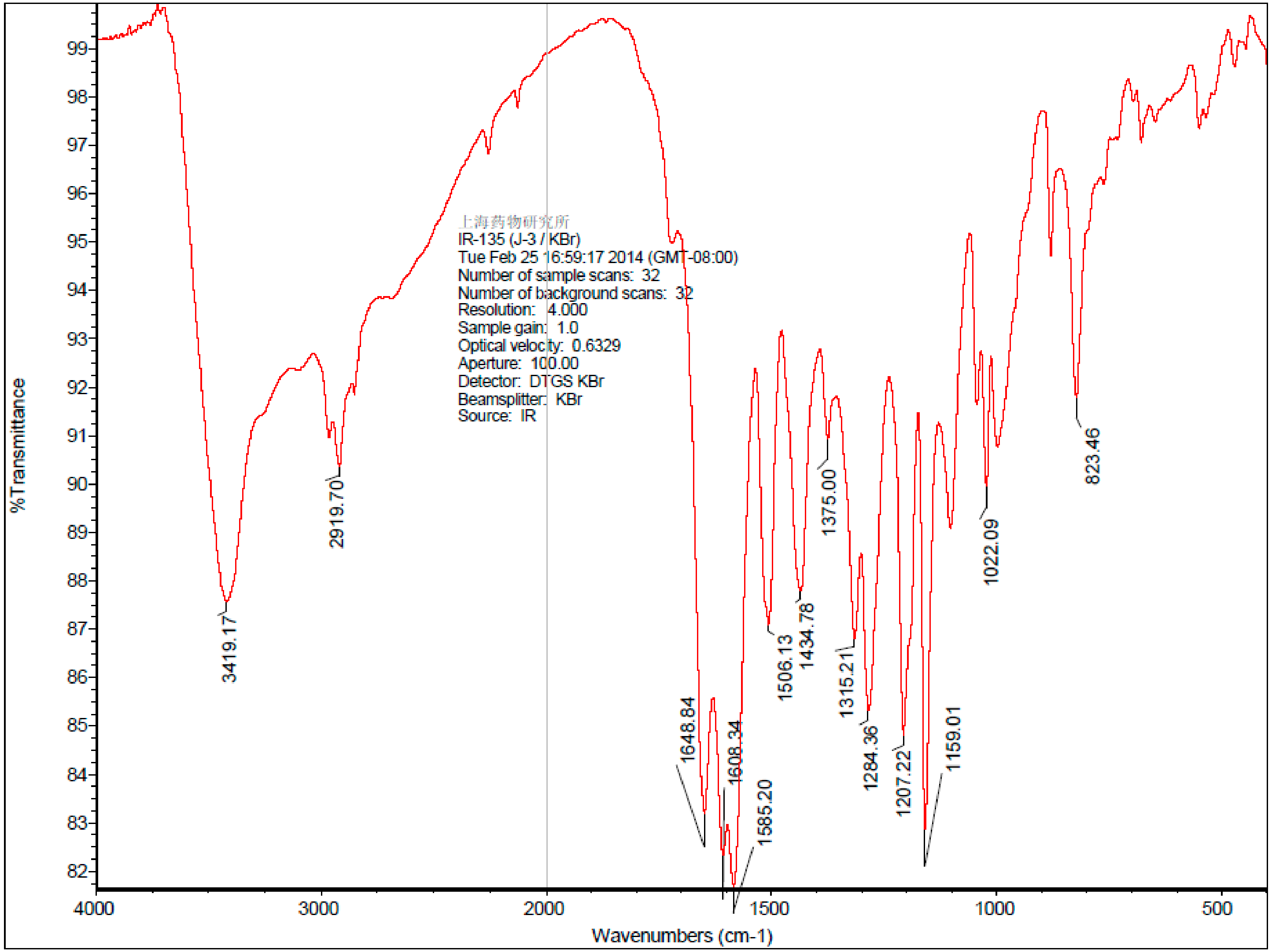

Figure S10. IR spectrum of **2**.

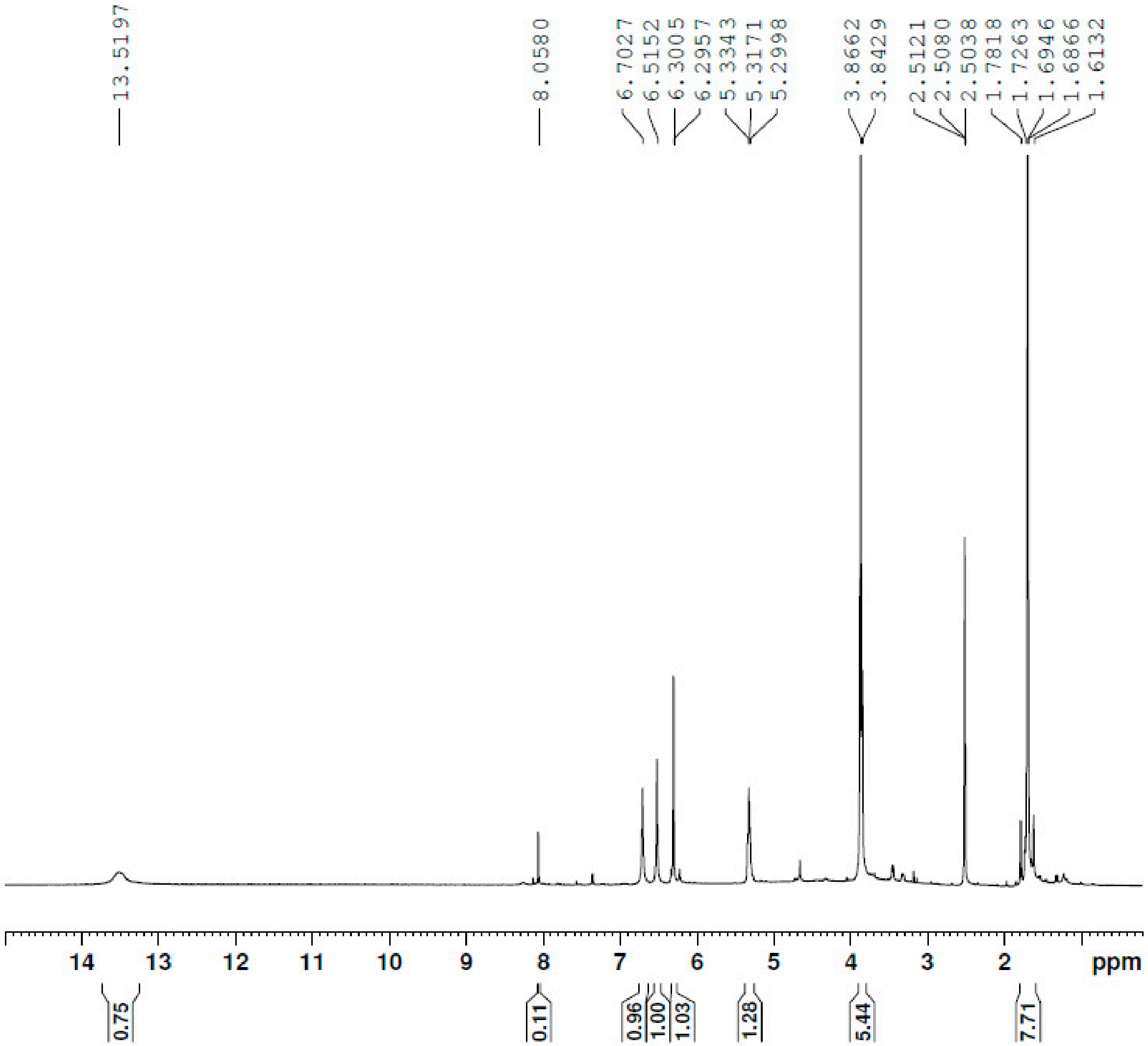

Figure S11. ^{1}H-NMR spectrum (400 MHz, DMSO-d_6) of **2**.

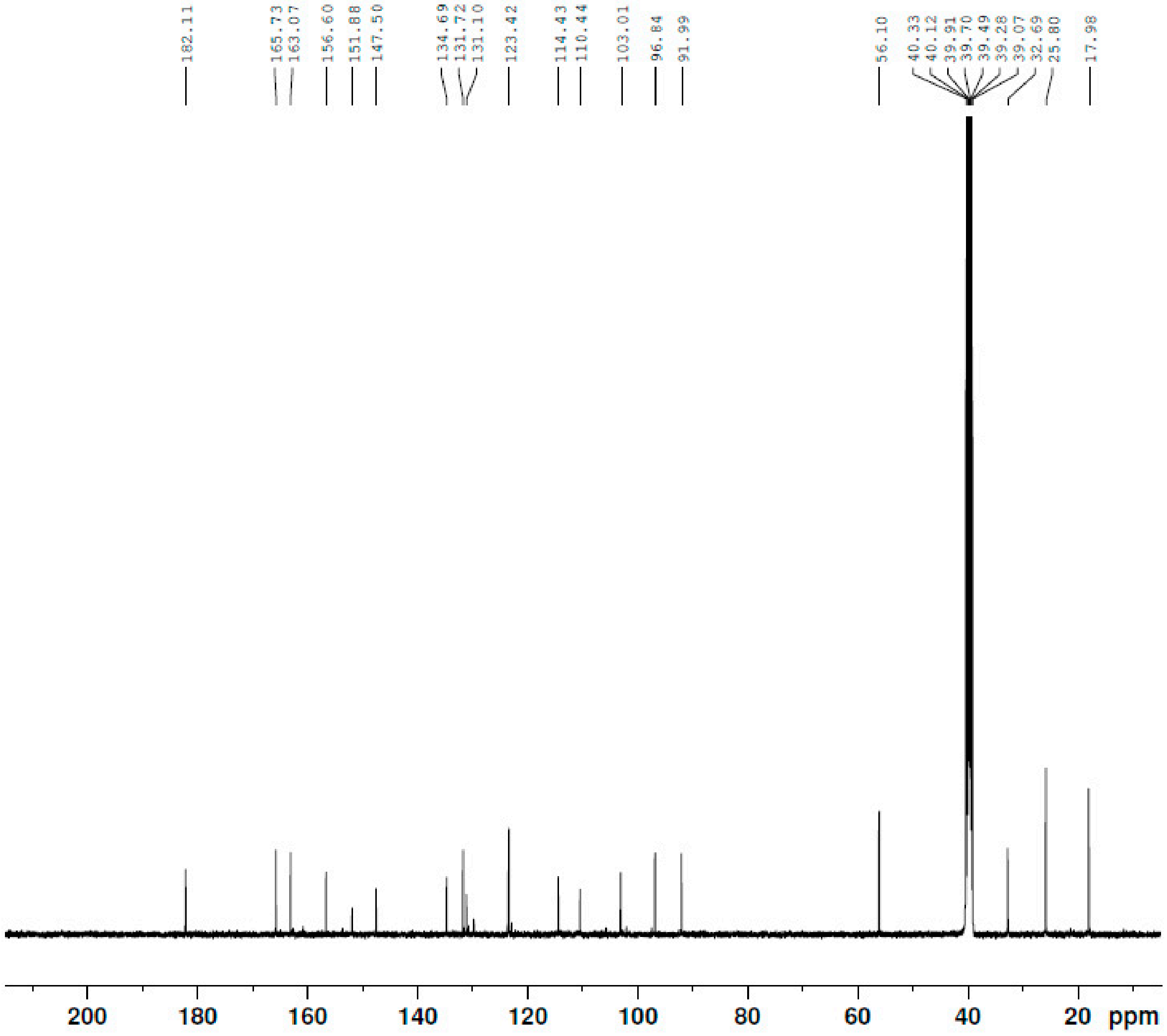

Figure S12. ^{13}C-NMR spectrum (100 MHz, DMSO-d_6) of **2**.

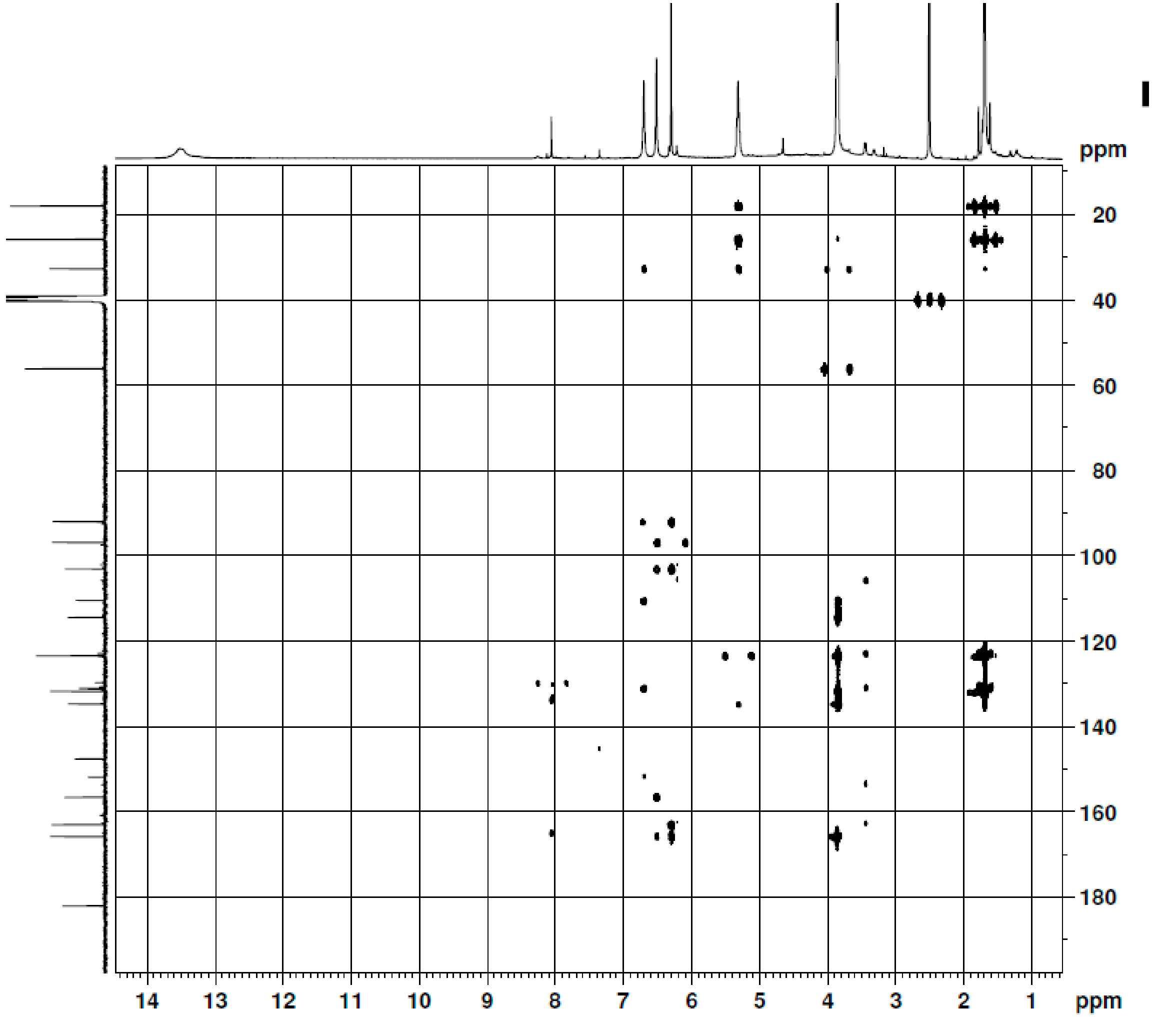

Figure S13. HMBC spectrum (400 MHz, DMSO-d_6) of **2**.

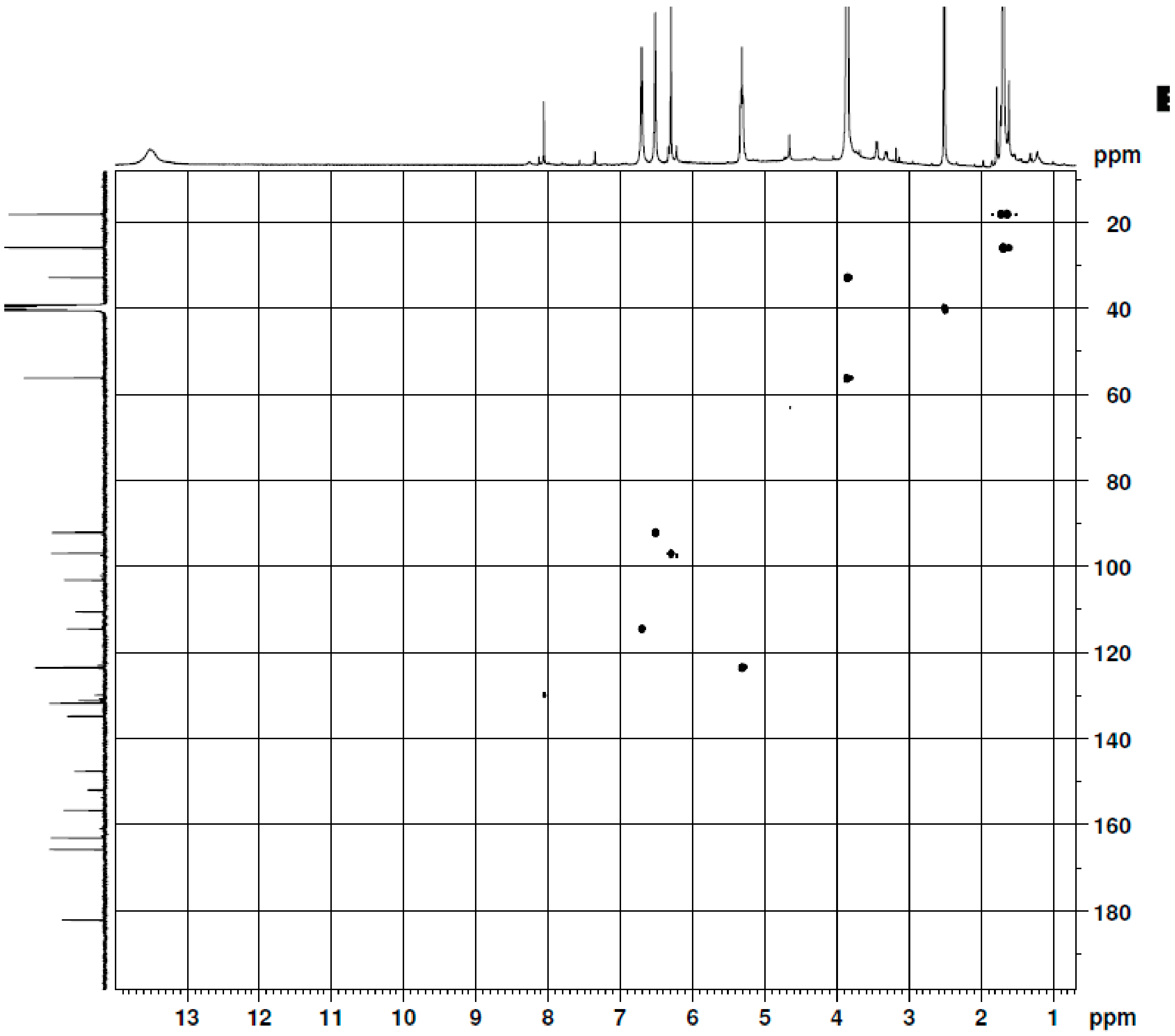

Figure S14. HSQC spectrum (400 MHz, DMSO-d_6) of **2**.

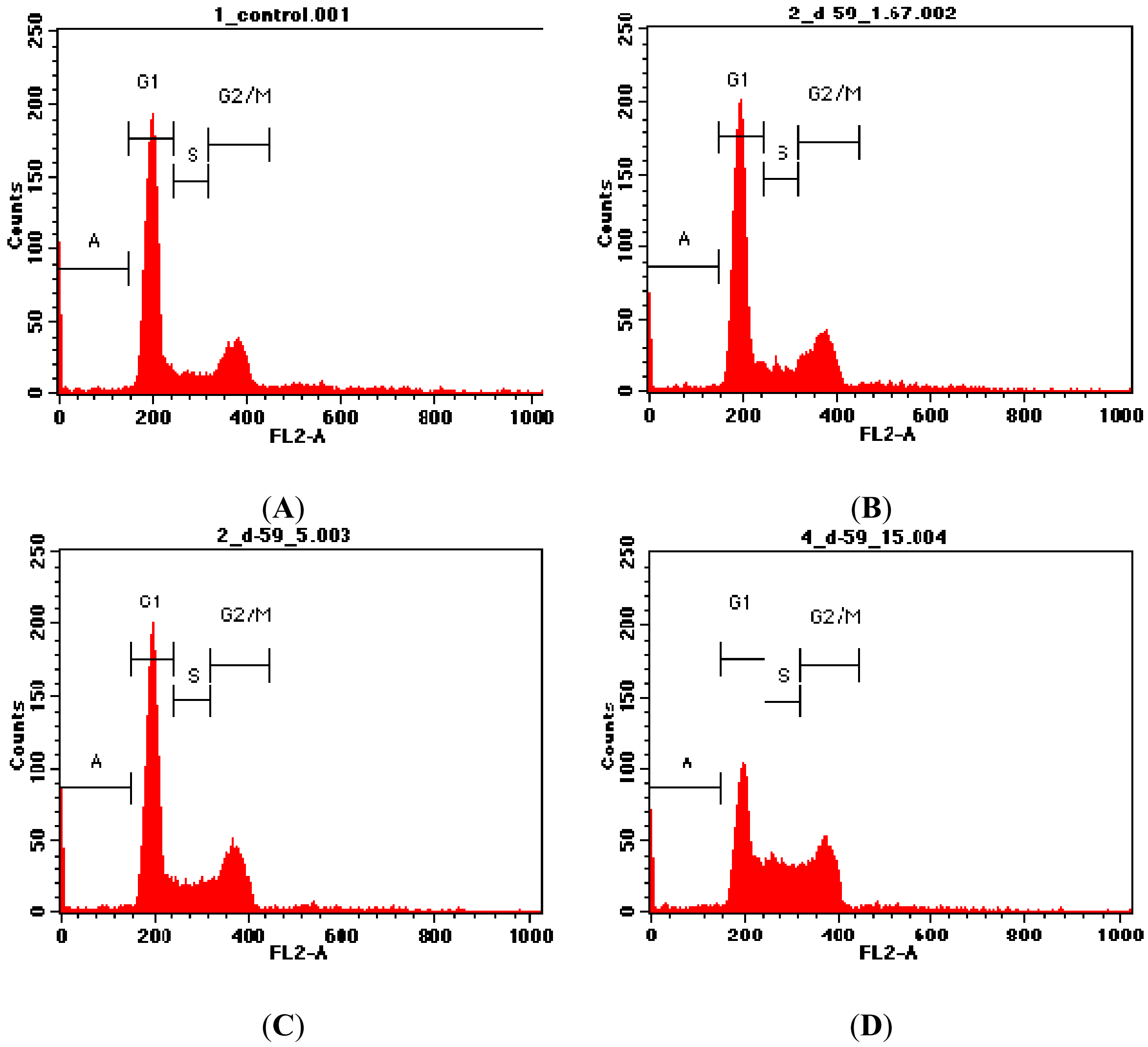

Figure S15. Flow cytometry analysis of HeLa cells. HeLa cells were treated with cisplatin or compound: control (**A**); 1.67 µM **3** (**B**); 5 µM **3** (**C**); 15 µM **3** (**D**) for 24 h.

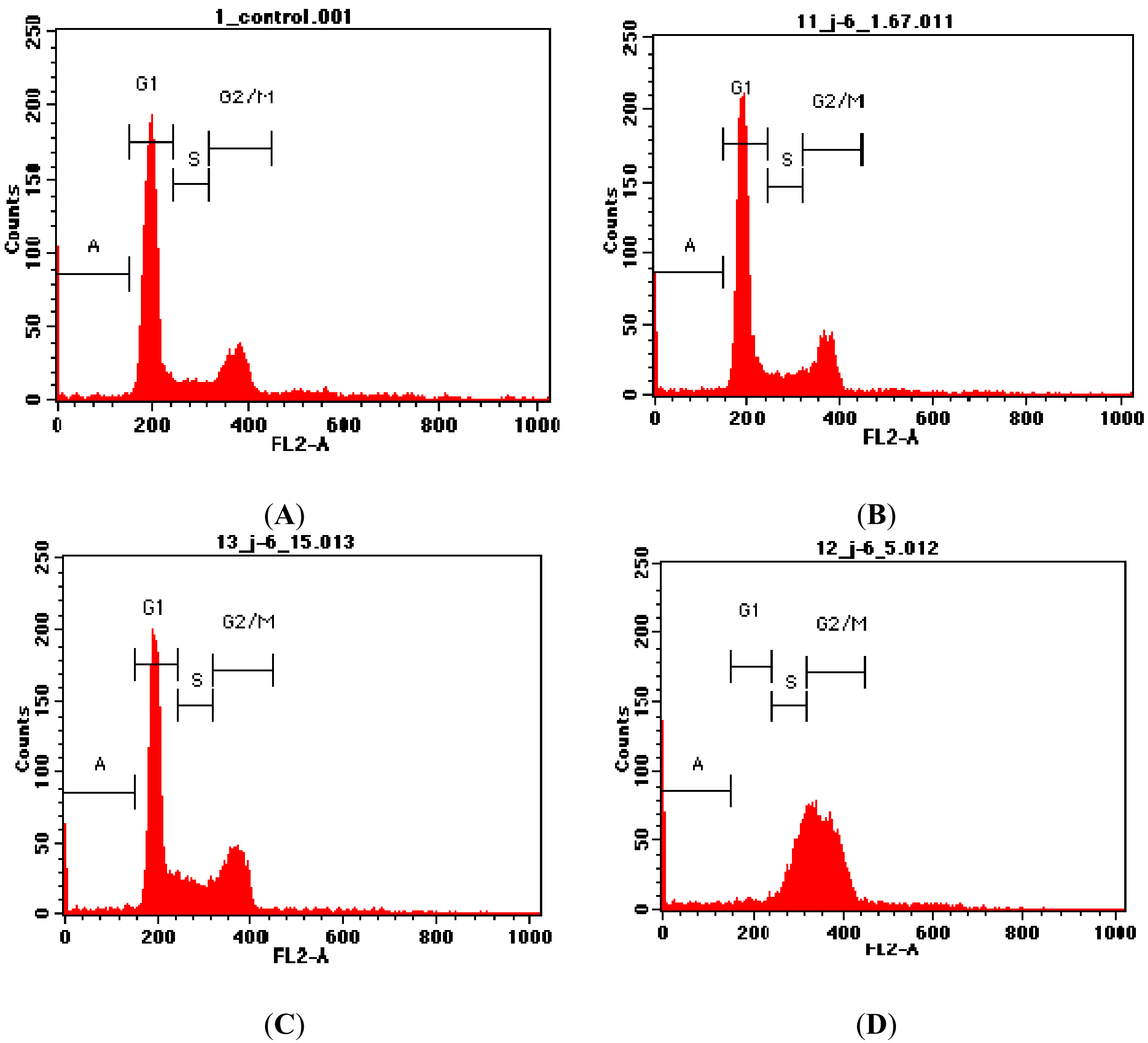

Figure S16. Flow cytometry analysis of HeLa cells. HeLa cells were treated with cisplatin or compound: control (**A**); 1.67 μM **4** (**B**); 5 μM **4** (**C**); 15 μM **4** (**D**) for 24 h.

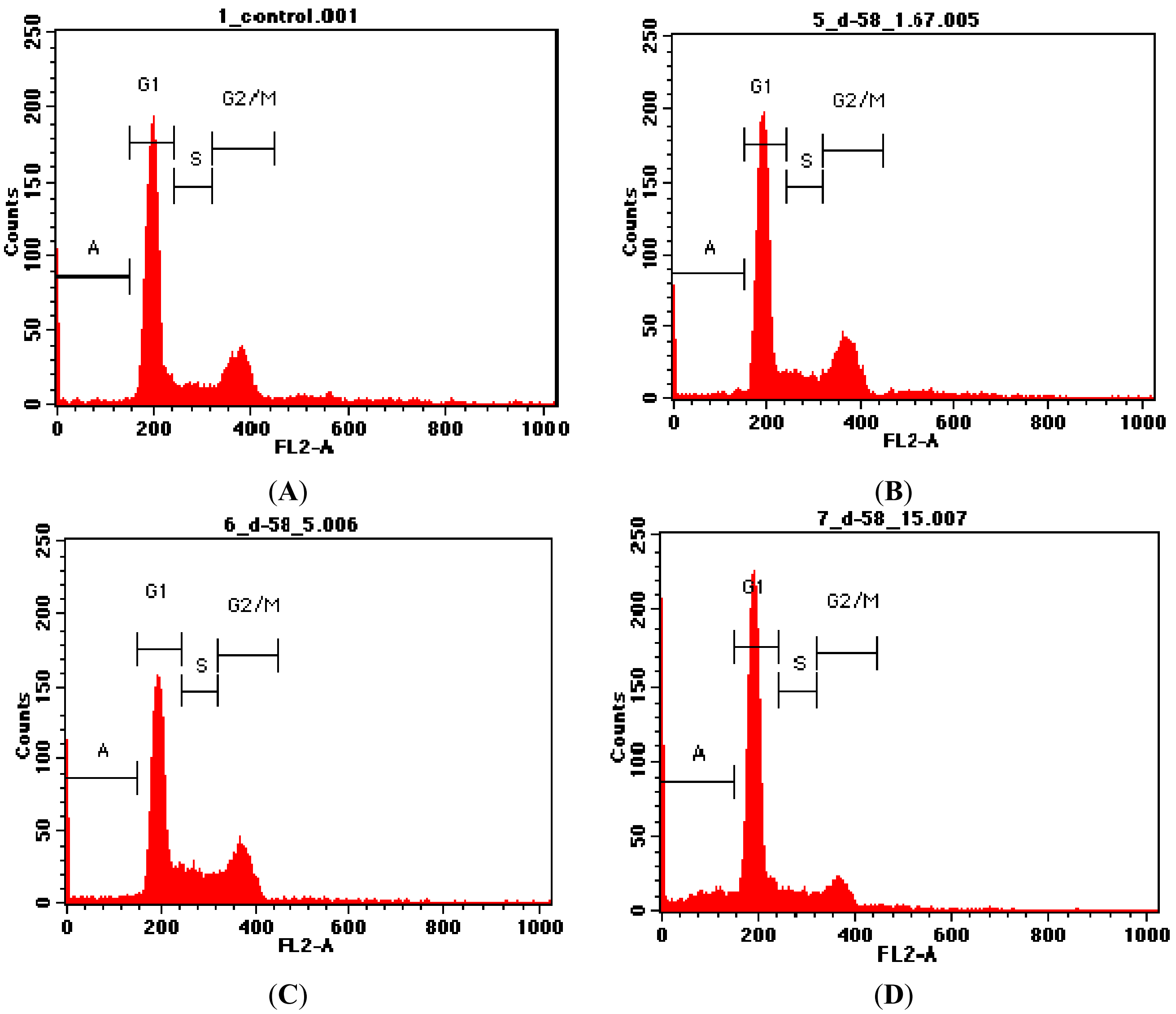

Figure S17. Flow cytometry analysis of HeLa cells. HeLa cells were treated with cisplatin or compound: control (**A**); 1.67 µM Xanthone V_1 (**B**); 5 µM Xanthone V_1 (**C**); 15 µM Xanthone V_1 (**D**) for 24 h.

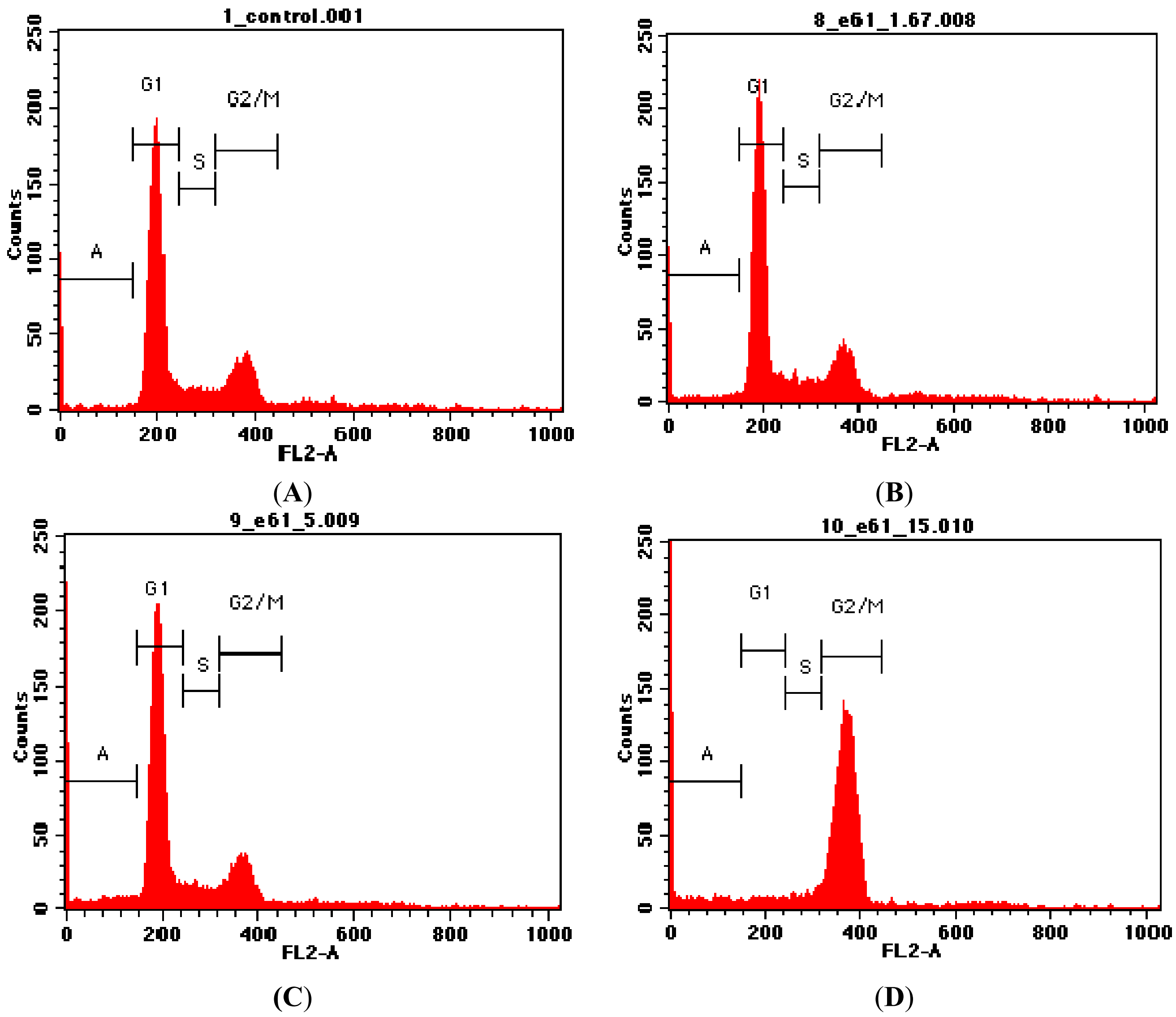

Figure S18. Flow cytometry analysis of HeLa cells. HeLa cells were treated with cisplatin or compound: control (**A**); 1.67 μM jacareubin (**B**); 5 μM jacareubin (**C**); 15 μM jacareubin (**D**) for 24 h.

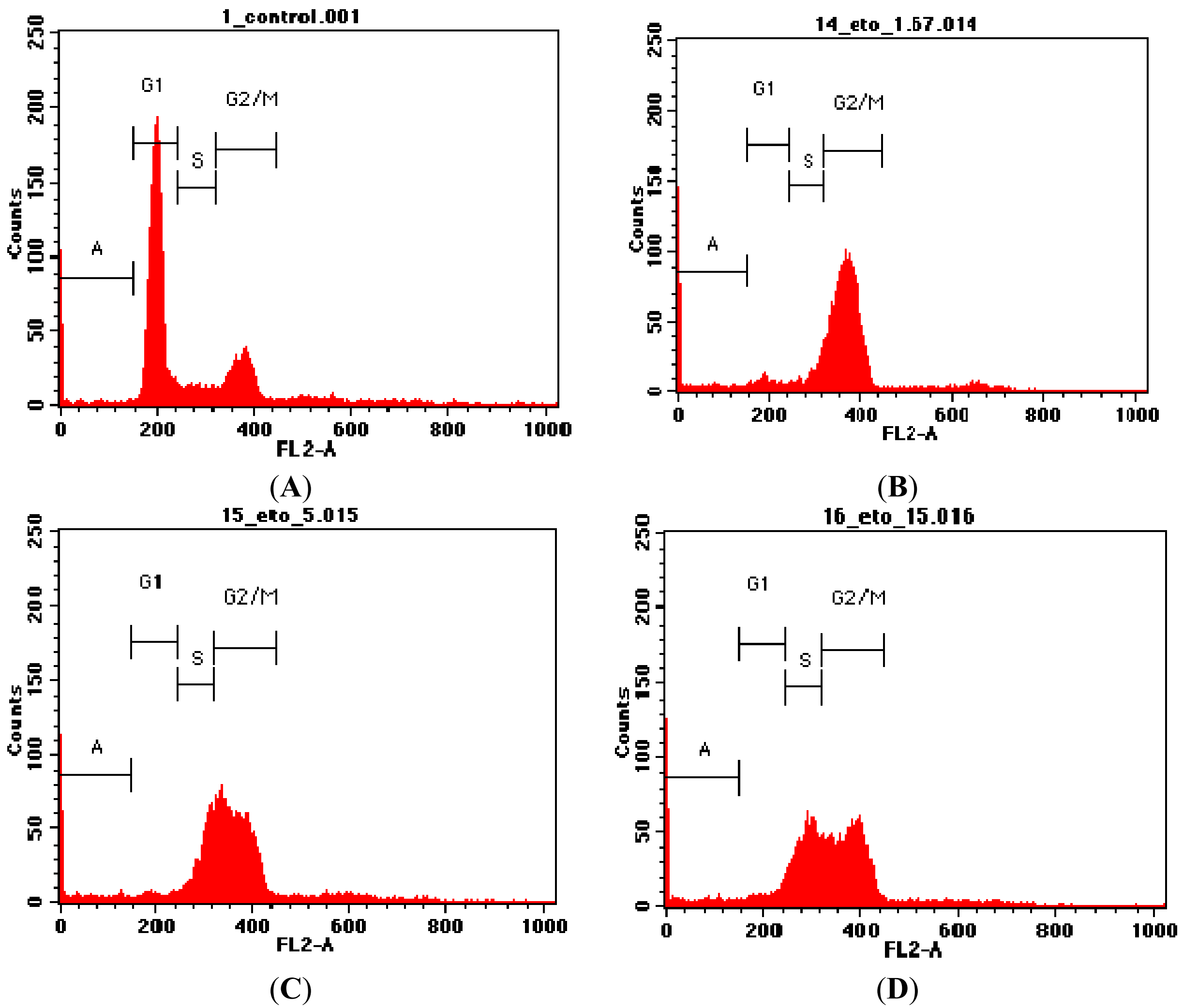

Figure S19. Flow cytometry analysis of HeLa cells. HeLa cells were treated with cisplatin or compound: control (**A**); 1.67 µM etoposide (**B**); 5 µM etoposide (**C**); 15 µM etoposide (**D**) for 24 h.
